# Do proton pump inhibitors alter the response to immune checkpoint inhibitors in cancer patients? A meta-analysis

**DOI:** 10.3389/fimmu.2023.1070076

**Published:** 2023-01-26

**Authors:** Sébastien Lopes, Lucile Pabst, Anne Dory, Marion Klotz, Bénédicte Gourieux, Bruno Michel, Céline Mascaux

**Affiliations:** ^1^ Pharmacy sterilization department, Nouvel Hopital Civil, Strasbourg University Hospital, Strasbourg, France; ^2^ Pulmonology department, Nouvel Hopital Civil, Strasbourg University Hospital, Strasbourg, France; ^3^ Laboratory Streinth (STress REsponse and INnovative THerapy against cancer), Inserm Unité Mixte de Recherche (UMR_S 1113), Interface de Recherche Fondamentale et Appliquée en Cancérologie (IRFAC), Université de Strasbourg, Instituts Thématiques Interdisciplinaires (ITI) InnoVec, Strasbourg, France

**Keywords:** immune checkpoint inhibitors, proton pump inhibitors, survival, solid cancer, meta – analysis

## Abstract

**Introduction:**

Gut microbiota can significantly affect the effectiveness of immune checkpoint inhibitors (ICIs) in cancer patients. Recently, antibiotics were shown to decrease survival rate of patients treated by ICIs. Proton pump inhibitors (PPIs) can indeed modulate microbiota’s diversity, therefore altering ICIs response. A meta-analysis was performed based on published data to verify this hypothesis.

**Methods:**

In this study, over 41 publications, exploring the impact of concomitant PPI treatment on outcomes of ICI-treated patients, were analyzed. Evaluated endpoints were overall survival (OS) and progression-free survival (PFS). Pooled hazard ratios (HRs) with a 95% confidence interval (CI) were reported in ICIs in PPI users versus non-PPI users. Subgroup analyses were performed to minimize the impact of study heterogeneity and to investigate the influence of PPI on the different groups of interest. There was no evidence of publication bias for OS and PFS analysis in subgroup analysis.

**Results:**

Forty-one studies were included in the meta-analysis, including a total of 20,042 patients. OS of patients receiving ICIs was negatively correlated in patients concomitantly treated with PPI (HR=1.37; 95%CI, 1.23–1.52). PFS of cancer patients receiving ICIs was also negatively correlated with PPI treatment (HR=1.28; 95%CI, 1.15–1.42). PPI and ICI use was associated with worst OS and PFS not only for non-small-cell lung cancer (NSCLC) or urothelial cancer patients but also for patients treated with anti PD-1 (OS) and anti PD-L1 (OS and PFS) immunotherapies when administered in non-first line and when PPI was received as baseline treatment or in 60 days before ICI initiation. PPI and ICI use also showed the worst OS and PFS for patients from Europe and Asia.

**Conclusion:**

This meta-analysis suggests that PPI treatment leads to significantly worse outcomes in advanced cancer patients treated by ICIs in terms of PFS and OS.

## 1 Introduction

Proton pump inhibitors (PPIs) are one of the most prescribed therapeutic classes in the world (1). The indications of PPIs are the treatment of gastroesophageal reflux disease (GERD) and esophagitis reflux. PPIs are also particularly efficient to treat patients at risk of gastrointestinal lesions by non-steroidal anti-inflammatories (and their prevention) and are used to treat gastroduodenal ulcer and eradicate *Helicobacter pylori* with concomitant antibiotics (2). However, PPIs are also often used off-label and sometimes for longer than recommended (3). PPIs also have multiple side effects. Possible short-term use side effects include rash, headache, dizziness, flatulence, abdominal pain, nausea, constipation and diarrhea, while possible long-term use may lead to enteric infection (particularly *Clostridium difficile* infection), peritonitis, liver diseases, pneumonia, ions and vitamins deficiencies (calcium, magnesium, iron, and vitamin B12), kidney disease, acute kidney injury, and finally dementia. Unnecessary or too long exposure can therefore present some risks for patients (4).

At the same time, the use of immune checkpoint inhibitors (ICIs) have revolutionized cancer patients’ treatment, particularly for non-small cell lung cancer (NSCLC) (5), melanoma (6), and urothelial carcinoma (7). The growing use of immunotherapy highlights the importance and risks of pharmacodynamics drug interactions. For example, recent publications showed the decrease in both the progression-free survival (PFS) and overall survival (OS) for patients with NSCLC treated by corticosteroids (8, 9). Such effect can be explained not only by the pharmacodynamics of corticosteroids and the inhibition of the inflammatory response and immune system homeostasis but also by their immunosuppressive proprieties in chronic uses, which is totally in opposition with action mechanism of ICIs.

In addition, antibiotics could be linked with poorer outcomes in cancer patients treated by ICIs. Several studies have shown a decrease in OS (10–13), PFS (12, 13), and disease control (10). Such pharmacodynamic interactions may be explained by the alteration of gut microbiota and a decrease in bacterial diversity by antibiotics treatment. In fact, ICI responses are closely related to the gut microbiome composition (14) because bacteria types or bacteria metabolites modulate the antitumor immunity and inflammation (15).

Other therapies can also modulate gut microbiota and therefore alter responses to ICIs therapies. Among those, PPIs are frequently prescribed in cancer patients, and several publications have shown that PPIs may be associated with poor outcome when used concomitantly with ICIs. However, this impact is still debated and a meta-analysis was therefore conducted to evaluate the role of the use of PPIs concomitantly with ICIs on the outcome of cancer patients.

## 2 Materials and methods

### 2.1 Identification of the publication for aggregation

For this systematic review of the literature on the role of PPIs on outcome with ICIs for cancer patients, the search for relevant publications was performed in both PubMed and Cochrane library database. Additional web searches were also performed to find other studies. The keywords and Medical Subject Headings (MeSH) used were “immunotherapy,” “immune checkpoint inhibitor,” “PDL-1 antibody,” “PD1-antibody,” “CTLA-4 inhibitor,” “pembrolizumab,” “atezolizumab,” “ipilimumab,” “nivolumab” “durvalumab,” “proton pump inhibitor,” “omeprazole,” “pantoprazole,” “rabeprazole,” “esomeprazole,” “lansoprazole,” “dexlansoprazole,” “concomitant medication,” “chronic medication,” “survival,” “overall survival,” and “progression free survival.”

Studies with various associations of previously cited keywords were included. Research strategy was as follows: (immunotherapy OR immune checkpoint inhibitor OR PDL-1 antibody OR PD1-antibody OR CTLA-4 inhibitor OR pembrolizumab OR atezolizumab OR ipilimumab OR nivolumab OR durvalumab) AND (proton pump inhibitor OR omeprazole OR pantoprazole OR rabeprazole OR esomeprazole OR lansoprazole OR dexlansoprazole OR concomitant medication OR chronic medication) AND (survival OR overall survival OR progression-free survival). Only studies with the following criteria were considered: 1) include patients diagnosed for advanced malignant tumors (any types of cancer) treated with ICIs, 2) ICIs could be administered alone or in combination with other anticancer drugs regardless of the therapeutic line, 3) potential association between PPI use and outcomes (OS and/or PFS) when co-prescribed with ICI needed to be assessed by comparing a PPI group and PPI-free group based on their historic use, and finally, 4) statistical data, in particular hazard ratio (HR), with 95% CI for OS and PFS were required.

Publications not responding to the previous criteria were discarded together with case reports and animal experiments. Reviews were not included in the analysis but considered by the authors to find potential missing publications. Studies that did not report informations of patients or did not present sufficient data on survival were excluded. Only full papers and abstracts with sufficient data were selected. Studies not yet fully published were not included in the meta-analysis. All reference lists from assessed articles were examined to identify additional potential articles of interest. The Preferred Reporting Items for Systematic Review and Meta-Analysis (PRISMA) guidelines were used to assess publications. Full papers published until November 2022 were included.

### 2.2 Quality evaluation

The Newcastle Ottawa quality assessment Scale (NOS) (16) was used for quality evaluation of the publications included in the meta-analysis. Each study was evaluated on three aspects: selection of groups (representativeness of the exposed cohort, selection of the non-exposed cohort, ascertainment of exposure, demonstration that the outcome of interest was not present at the start of the study), and group’s comparability and ascertainment of outcomes (assessment of outcome, sufficient follow-up for the occurrence of outcomes, and adequacy of follow-up cohort). Two investigators (SL and MK) graded independently all the studies with a maximum of 9 points. The selection of patients, assessment of outcomes, and comparability were assessed. The studies with less than six stars were excluded from the meta-analysis. The two investigators were used to score studies independently to avoid any bias. When discordant scores were obtained, the study was revalued, and a consensus was found after discussion between the authors.

### 2.3 Data extraction

Two reviewers independently assessed the eligibility of the studies included in the meta-analysis. One investigator (LP), with experience in statistical analysis, extracted the following data from the studies: first author, publication year, number of PPI and non-PPI users, hazard ratio (HR), and 95%CI for PFS and/or OS between PPI users and non-users. Another investigator (SL) was responsible for extracting the following data: type of studies, number of patients included, patients age and sex, Eastern Cooperative Group performance status (ECOG PS), region, cancer type, treatment line, ICI treatment used, type of PPI treatment, PPI use window, and outcomes.

### 2.4 Statistical analysis

The overall HRs and 95%CI for PFS and OS were calculated to compare the impact of PPI use on ICI treatment. An HR > 1.0 indicated a better outcome in the no-PPI-treated arm. On the other hand, an HR < 1.0 implied a greater treatment effect in the PPI-treated arm and therefore a favorable effect of PPI use on ICI treatment. Statistical significance was determined using the Cochran’s chi-square test. p-values of <0.05 were considered statistically significant.

The heterogeneity of the included studies was estimated using Cochrane’s Q test and I² statistics. For the Q test, p<0.10 represented statistically significant heterogeneity. When I² was higher than 50%, substantial heterogeneity was considered between studies. For low heterogeneity studies (p<0.10 and I² ≤50%), a fixed-effects model was used, while for high-heterogeneity studies (p<0.10 and I²>50%), a random-effects model was used. Subgroup analyses were performed in order to minimize the impact of study heterogeneity and therefore to investigate whether the influence of PPI use varied between different groups of interest (region, type of cancer, type of ICI, treatment line, and PPI-use window).

Sensitivity analyses were performed using the “one-study removed” approach to assess its effect on the pooled outcome hazard ratio.

Funnel plots with Egger’s regression tests were used to examine publication bias across studies.

All statistical analyses and forest plots were conducted using Review Manager (RevMan 5.4; the Cochrane Collaboration, Oxford, United Kingdom).

## 3 Results

### 3.1 Search results

A total of 2,995 publications were collected from the primary publication search, from which 2,919 were immediately discarded because they dealt with different topics or animal studies. A total of 71 studies were included in the full text analyze. Of these, 30 were excluded for the following reasons: 10 were reviews and/or meta-analysis, 11 were not relevant because of insufficient data on the outcomes or because hazard ratio was not available, 8 were non-PPI studies, and 1 study included the same patient cohort as another publication. Finally, 41 publications were eligible and were included in the meta-analysis (17, 18, 19; 20, 21, 10, 22-25, 26; 27-38, 39; 40-43, 44; 45-55, 13).

**Figure 1:**
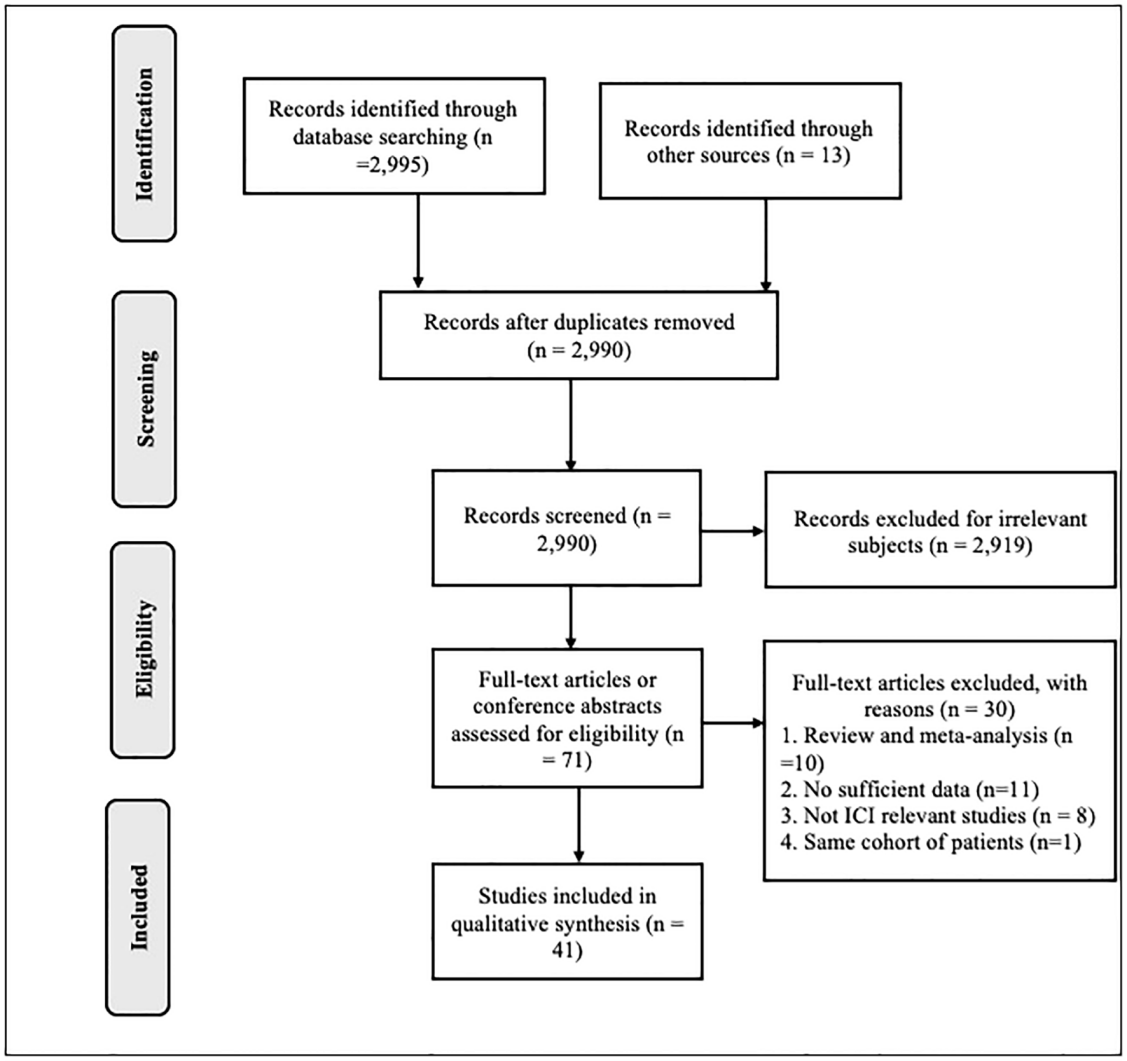
Preferred Reporting Items for Systematic Review and Meta-Analysis (PRISMA) flow diagram of articles identification and selection. PPI, proton pump inhibitor.

The PRISMA flow diagram of articles identification and selection is shown in **Figure 1**.

### 3.2 Baselines characteristics of the included publications

Baseline characteristics of the included publications are shown in **Table 1**. All of them were retrospective studies (**Table 2**), and they included 20,042 patients in total. Among these patients, 8,647 (43,1%) had taken PPI before (30, 60, or 90 days prior to initiate ICIs), during, and/or after (30–60 days) the immunotherapy treatment. The most common cancer observed in these studies was NSCLC with 11,555 cases (57,7%). All the publications assessed the impact of PPI use on OS and/or PFS, with 2 studies showing a positive impact, 21 a negative impact, and 18 no significant effect of PPI. All HR values were extracted from either univariate or multivariate analysis (if available). The agents used for immunotherapy treatments were anti-PD-1, anti-PD-L1, and anti-CTLA-4 for first line, second line, or beyond.

**Table 1:** Baseline characteristics of included publications.

Author	Year	Patient number	Region	Male (%)	Median age	ECOG PS 0-1/2	Cancer type	ICI treatment	Treatment line	ICI monotherapy or association	PPI	No PPI	PPI treatment	PPI use window
**Afzal** (17)	2019	120	America	NA	65	NA	Melanoma	Pembrolizumab, nivolumab, ipilimumab	NA	Monotherapy or association	29	91	Omeprazole (majority)	At the start of ICI
**Araujo** (18)	2021	216	America	NA	59	199/17	Multiple	Anti PD1, anti PD-L1	NA	NA	57	NA	NA	60 days prior ICIs initiation
**Baeck** (19)	2022	1,646	Asia	1,323 (80)	66	NA	NSCLC	Pembrolizumab, nivolumab, atezolizumab	Second or beyond	NA	823	823	NA	Within 30 days prior ICIs initiation
**Buti** (20)	2021	217	Europe	148 (68,2)	69	189/28	Multiple	Anti-PD-1, anti PD-L1, anti-CTLA-4	First, second, or beyond	Monotherapy	104	113	NA	NA
**Castro Balado** (21)	2021	49	Europe	37 (75,5)	66	NA	NSCLC	Pembrolizumab	First	Monotherapy	26	23	NA	NA
**Chalabi** (10)	2020	757	America/Europe	471 (62,2)	NA	755/NA	NSCLC	Atezolizumab	Second or beyond	Monotherapy	234	523	Omeprazole (majority)	Within 1 month before or after ICIs initiation
**Conde-Estevez** (22)	2021	70	Europe	53 (75,7)	66	62/8	NSCLC	Atezolizumab, pembrolizumab, nivolumab	Second or beyond	Monotherapy	59	11	NA	NA
**Cortellini** (23)	2020	1,012	Europe	647 (63,9)	69	870/142	Multiple	Nivolumab, pembrolizumab, atezolizumab	First, second, or beyond	Monotherapy	491	521	NA	NA
**Cortellini** (24)	2021	950	Europe	625 (65,8)	70	785/165	NSCLC	Pembrolizumab	First	Monotherapy	474	476	NA	NA
**Failing** (25)	2016	80	America	NA	58	NA	Melanoma	Ipilimumab	First	Monotherapy	17	63	Omeprazole (majority), pantoprazole, esomeprazole, lansoprazole	At the start of ICI
**Fukuokaya** (26)	2022	227	Asia	165 (72,7)	70	NA	Urothelial carcinoma	Pembrolizumab	Second or beyond	Monotherapy	86	141	Esomeprazole, lansoprazole, omeprazole, rabeprazole, vonoprazan	Within 1 month before or after ICIs initiation
**Gaucher** (27)	2021	372	Europe	244 (65,6)	64	295/77	Multiple	Ipilimumab, nivolumab, pembrolizumab	First, second, or beyond	Monotherapy or association	149	223	NA	At the ICI initiation or in the following 60 days
**Giordan** (28)	2021	212	Europe	143 (68)	64	161/50	Multiple	Nivolumab, pembrolizumab, ipilimumab	First, second, or beyond	Monotherapy or association	74	138	Omeprazole, pantoprazole, esomeprazole, lansoprazole, rabeprazole	Within 30 days prior ICIs initiation
**Hakozaki** (29)	2018	90	Asia	57 (63,3)	67	64/26	NSCLC	Nivolumab	Second or beyond	Monotherapy	47	43	NA	Within 30 days prior ICIs initiation
**Homicsko** (30)	2022	1,505	Majority America/Europe	951 (63,2)	NA	1,498/7	Melanoma	Nivolumab, ipilimumab	First	Monotherapy or association	291	1,214	Omeprazole, pantoprazole, esomeprazole, lansoprazole, rabeprazole, dexlansoprazole	Within 30 days prior ICIs initiation
**Hopkins** (31)	2020	1,360	America/Europe	NA	68	1,336/24	Urothelial carcinoma	Atezolizumab	First, second	Monotherapy	471	889	Omeprazole, pantoprazole, esomeprazole, lansoprazole, rabeprazole, dexlansoprazole	Within 30 days prior or after ICIs initiation
**Hopkins** (32)	2021	1,202	Worldwide	720 (59,9)	63	1,202/0	NSCLC	Atezolizumab	First	Association	441	761	Omeprazole, pantoprazole	Within 30 days prior or after ICIs initiation
**Hossain** (33)	2020	63	Oceania	NA	NA	NA	NSCLC	NA	NA	NA	34	29	NA	30 days after ICIs initiation
**Husain** (34)	2021	1,091	America	647 (59,3)	62	813/184	Multiple	Anti PD1, anti PD-L1, anti CTLA-4, other	First, second, or beyond	NA	415	676	NA	At the same time of ICI
**Iglesias-Santamaria** (35)	2019	102	Europe	84 (82,2)	66	91/4	Multiple	Ipilimumab, nivolumab, pembrolizumab, atezolizumab	First, second, or beyond	NA	78	23	NA	NA
**Jun** (36)	2021	314	America/Europe/Asia	248 (79)	66	NA	Hepatocellular carcinoma	Anti PD-1, anti CTLA-4	First, second, or beyond	Monotherapy or association	85	229	Omeprazole, pantoprazole esomeprazole, lansoprazole Rabeprazole dexlansoprazole	Within 30 days prior ICI initiation
**Kostine** (37)	2021	634	Europe	443 (70)	65	528/98	Multiple	Anti-CTLA-4, anti-PD1, anti-PD-L1	First or beyond	NA	239	396	Omeprazole, pantoprazole, lansoprazole, rabeprazole	Within 30 days before or after ICIs initiation
**Kulkarni** (38)	2019	203	Europe	NA	NA	NA	Multiple	Anti-PD-1, anti-PD-L1	NA	NA	74	129	NA	Within 1 month before ICIs initiation
**Kunimitsu** (39)	2022	79	Asia	59 (74,7)	72	56/23	Urothelial carcinoma	Pembrolizumab	First, second, or beyond	Monotherapy	45	34	Lansoprazole, esomeprazole, rabeprazole, vonoprazan	within 60 days prior and/or 30 days after treatment initiation
**Miura** (40)	2021	300	Asia	226 (75,3)	65	246/54	NSCLC	Nivolumab, pembrolizumab	First, second, or beyond	Monotherapy	163	137	NA	NA
**Mollica** (41)	2021	219	America/Europe	155 (71)	61	NA	Renal cell carcinoma	Nivolumab, ipilimumab	First, second, or beyond	Monotherapy or association	113	106	NA	30 days before ICIs initiation
**Nguyen** (42)	2019	95	Asia	62 (65,3)	68	NA	Multiple	Nivolumab	NA	Monotherapy	40	55	NA	2 weeks during the ICI administration
**Okuyama** (43)	2022	155	Asia	109 (70,3)	72	121/34	Urothelial carcinoma	Pembrolizumab, nivolumab, atezolizumab, and durvalumab	Second or beyond	NA	99	56	NA	Within 30 days before or during the ICI therapy
**Peng** (44)	2022	233	America	130 (55,8)	64	186/47	Multiple	Nivolumab, pembrolizumab, and ipilimumab	First, second, or beyond	Monotherapy or association	89	144	NA	Within 30 days before or after ICIs initiation
**Perez-Ruiz** (45)	2020	253	Europe	176 (70)	61	216/31	Multiple	Anti-CTLA-4, anti-PD-1	First, second, or beyond	NA	135	118	NA	Within 60 days prior to 30 days after
**Rassy** (46)	2022	707	Europe	546 (72,2)	64	568/103	Renal cell carcinoma	Nivolumab	Second or beyond	Monotherapy	196	511	NA	At the same time of ICI
**Routy** (47)	2017	249	Europe	177 (71,1)	63	NA	Multiple	Anti-PD-1, anti-PD-L1, anti-CTLA-4	First, second, or beyond	Monotherapy or association	187	62	NA	Within 60 days prior to 30 days after
**Ruiz-Banobre** (48)	2021	119	Europe	96 (81)	69	99/20	Urothelial carcinoma	Atezolizumab, pembrolizumab, nivolumab, durvalumab	First, second, or beyond	Monotherapy	54	65	NA	Within 30 days prior ICI initiation
**Spakowicz** (49)	2020	689	America	402 (58.3)	62	457/139	Multiple	Atezolizumab, pembrolizumab, nivolumab, durvalumab and tremelimumab	NA	NA	255	434	NA	Within 30 days prior ICI initiation
**Stein** (50)	2021	232	Europe	NA	NA	NA	Melanoma	Nivolumab, pembrolizumab	First or beyond	NA	86	146	NA	At the same time of ICI
**Stokes** (51)	2021	3,634	America	3,525 (97)	69	NA	NSCLC	Nivolumab, pembrolizumab, durvalumab, and atezolizumab	NA	NA	2,159	1,475	Omeprazole (majority)	Within 90 days ICIs initiation
**Svaton** (52)	2020	224	Europe	133 (59,3)	67	220/4	NSCLC	Nivolumab	First, second, or beyond	NA	64	160	Omeprazole, pantoprazole lansoprazole	Within 30 days before or after ICIs initiation
**Takada** (53)	2022	95	Asia	78 (82,1)	69	89/6	NSCLC	Nivolumab, pembrolizumab, and atezolizumab	NA	Monotherapy or chemotherapy combination	37	58	Esomeprazole, lansoprazole Rabeprazole, omeprazole, vonoprazan	the same time of ICI
**Tomita** (54)	2022	118	Asia	99 (83,8)	68	52/20	NSCLC	Nivolumab, pembrolizumab, and atezolizumab	First, second, or beyond	Monotherapy or chemotherapy combination	72	46	Esomeprazole, lansoprazole Rabeprazole, omeprazole, vonoprazan	Within 30 days before or after ICIs initiation
**Tomizaki** (55)	2022	40	Asia	30 (75)	72	NA	Urothelial carcinoma	Pembrolizumab	Second or beyond	Monotherapy	15	25	NA	Within 60 days before or 30 days after ICIs initiation
**Zaho** (13)	2019	109	Asia	89 (81,7)	62	107/2	NSCLC	Pembrolizumab, nivolumab, and camrelizumab	First, second, or beyond	Monotherapy or chemotherapy combination	40	69	NA	Within 1 month before or after ICIs initiation

PPI, proton pump inhibitor; ICI, immune checkpoint inhibitor; NA, not available; ECOG PS, Eastern cooperative group performance status; NSCLC, non-small cell lung cancer; ICI, immune checkpoint inhibitor; PD-1, programmed cell death protein 1; PD-L1, programmed cell death ligand 1; CTLA-4, cytotoxic T-lymphocyte-associated antigen 4.

**Table 2:** Outcomes data and NOS score of included publications.

Author	Year	Type of study	Outcomes	OS HR (95% CI)	PFS HR (95% CI)	NOS score
**Afzal**	2019	Retrospective	OS and PFS	1.01 (0.4-2)	0.3 (0.1-0.7)	6
**Araujo**	2021	Retrospective	OS, PFS	1.73 (1.23-2.44)	2.36 (1.67-3.34)	7
**Baeck**	2021	Retrospective	OS	1.64 (1.25-2.17)	NA	8
**Buti**	2021	Retrospective	OS and PFS	1.57 (1.13-2.18)	NA	6
**Castro Balado**	2021	Retrospective	OS and PFS	0.4 (0.17-0.93)	0.98 (0.43-2.21)	7
**Chalabi**	2020	Retrospective	OS and PFS	1.45 (1.2-1.75)	1.3 (1.1-1.53)	9
**Conde-Estevez**	2021	Retrospective	PFS	NA	2.91 (0.88-9.65)	6
**Cortellini**	2020	Retrospective	OS and PFS	1.26 (1.04-1.52)	1.26 (1.07-1.48)	8
**Cortellini**	2021	Retrospective	OS and PFS	1.49 (1.26-1.77)	1.32 (1.13-1.54)	7
**Failing**	2016	Retrospective	OS and PFS	0.44 (0.17-1.15)	0.6 (0.34-1.06)	8
**Fukuokaya**	2021	Retrospective	OS and PFS	2.02 (1.28 – 3. 18)	1.7 (1.23-2.35)	8
**Gaucher**	2021	Retrospective	OS	0.8 (0.6-1.08)	NA	7
**Giordan**	2021	Retrospective	OS and PFS	1.89 (1.29-2.9)	1.51 (1.11-2.05)	8
**Hakozaki**	2018	Retrospective	OS and PFS	1.9 (0.8-4.51)	NA	6
**Homicsko**	2022	Retrospective	OS and PFS	CheckMate 069: 2 (0.94-4.26) CheckMate 067: 0.9 (0.57-1.42) CheckMate 066: 1.07 (0.67-1.7)	CheckMate 069: 2.17 (1.1-4.25) CheckMate 067: 1.03 (0.7-1.52) CheckMate 066: 1.13 (0.74-1.17)	8
**Hopkins**	2020	Retrospective	OS and PFS	1.52 (1.27-1.83)	1.38 (1.18-1.62)	8
**Hopkins**	2021	Retrospective	OS and PFS	1.53 (1.21-1.95)	1.34 (1.12-1.61)	8
**Hossain**	2020	Retrospective	OS and PFS	1.66 (0.81-3.42)	1.34 (1.12-1.61)	6
**Husain**	2021	Retrospective	OS	1.99 (1.15-3.45)	NA	7
**Iglesias-Santamaria**	2019	Retrospective	OS and PFS	0.79 (0.4-1.56)	0.75 (0.42-1.34)	8
**Jun**	2021	Retrospective	OS	1.14 (0.84-1.54)	NA	7
**Kostine**	2021	Retrospective	OS and PFS	1.7 (1.4-2.08)	1.37 (1.12-1.66)	7
**Kulkarni**	2019	Retrospective	OS and PFS	NSCLC: 1.55 (1 - 2.4) Renal cell carcinoma: 1.01 (0.39 - 2. 62)	NSCLC: 1.15 (0.79 - 1.66) Renal cell carcinoma: 1.03 (0.53 - 1.97)	6
**Kunimitsu**	2022	Retrospective	OS and PFS	0.8 (0.4-1.56)	1.44 (0.79-2.6)	8
**Miura**	2021	Retrospective	OS	1.36 (0.96-1.91)	NA	8
**Mollica**	2021	Retrospective	OS and PFS	Nivolumab + ipilimumab: 1.12 (0.38 - 3.27) Nivolumab: 0.81 (0.53 - 1.24)	Nivolumab + ipilimumab: 1.04 (0.49 - 2.2) Nivolumab: 1.05 (0.73 - 1.5)	8
**Nguyen**	2019	Retrospective	OS and PFS	1.51 (0.87 - 2.6)	1.29 (0.8 - 2.07)	6
**Okuyama**	2022	Retrospective	OS and PFS	1.78 (1.03-3.07)	1.72 (1.07-2.77)	7
**Peng**	2022	Retrospective	OS and PFS	1.22 (0.8-1.96)	1.05 (0.76-1.45)	8
**Perez-Ruiz**	2020	Retrospective	OS	2.6 (1.6-4.22)	NA	7
**Rassy**	2022	Retrospective	OS and PFS	1.24 (0.98-1.58)	0.89 (0.74-1.08)	6
**Routy**	2017	Retrospective	OS and PFS	1.15 (0.87 - 1.53)	1.12 (0.83 - 1.51)	6
**Ruiz-Banobre**	2021	Retrospective	OS and PFS	1.83 (1.11-3.02)	1.94 (1.22-3.09)	9
**Spakowicz**	2020	Retrospective	OS	0.99 (0.85-1.16)	NA	6
**Stein**	2021	Retrospective	OS	1.83 (1.2-2.78)	NA	8
**Stokes**	2021	Retrospective	OS	0.96 (0.89-1.04)	NA	7
**Svaton**	2020	Retrospective	OS and PFS	0.822 (0.487-1.388)	0.737 (0.485-1.121)	8
**Takada**	2022	Retrospective	OS and PFS	2.55 (1.31-4.99)	4.12 (2.28-7.46)	7
**Tomita**	2022	Retrospective	OS	2.47 (1.28-4.74)	NA	7
**Tomizaki**	2022	Retrospective	OS and PFS	4.0 (1.22-13.15)	3.36 (1.17-9.60)	7
**Zaho**	2019	Retrospective	OS and PFS	1.47 (0.7-3.06)	1.1 (0.65-1.85)	8

OS, overall survival; PFS, progression-free survival; HR, hazard ratio; NOS, Newcastle–Ottawa Quality Assessment Scale; CI, confidence interval.

### 3.3 Quality assessment

All publications were graded with six to eight stars, on a maximum of 9 points, and no studies were excluded from the meta-analysis. The studies and their scores are shown in **Table 2**.

### 3.4 Effect of concomitant use of PPI on overall survival and progression-free survival

Among the 41 publications selected for the meta-analysis, 42 cohorts (the study from Homicsko et al. including three cohorts) provided data for OS (n=19,972 patients), and 31 cohorts provided data for PFS (n=11,086 patients).

**Figure 2:**
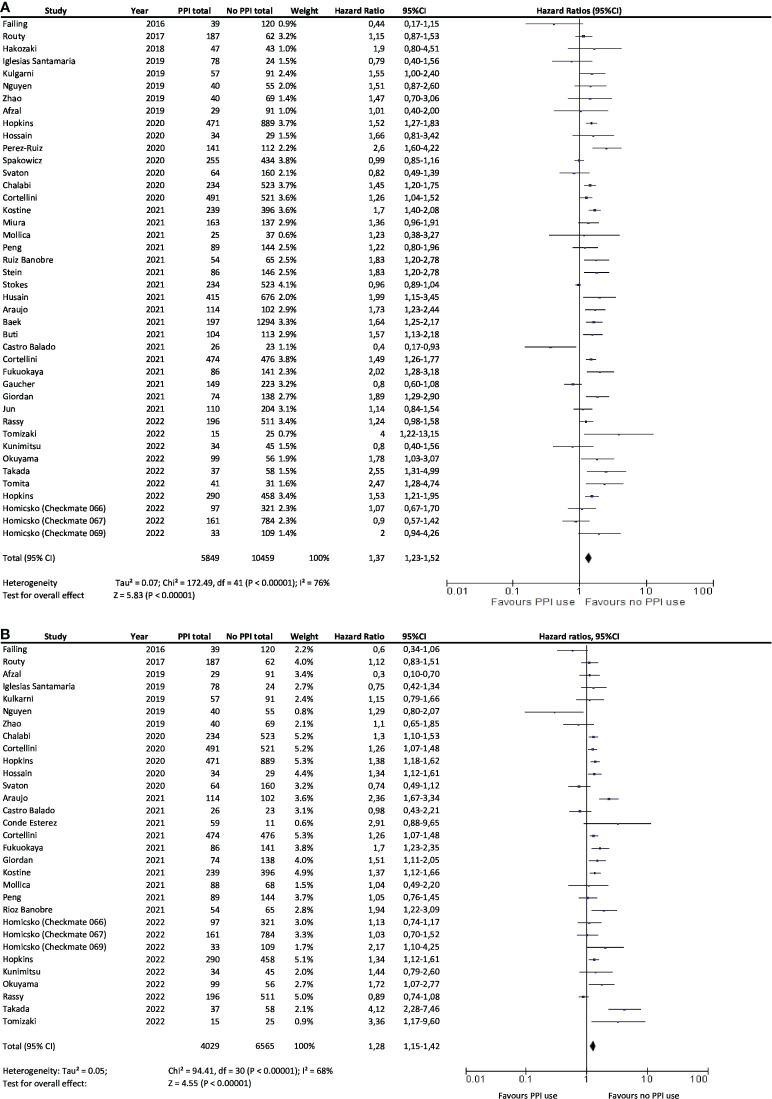
Association between PPI use and overall survival **(A)** and progression-free survival **(B)** in cancer patients treated with immunotherapy. PPI, proton pump inhibitor; CI, confidence interval.

A statistically significant association between PPI and ICI use and shorter OS was observed in 19 cohorts. No difference in OS was observed for patients treated with ICI whether they received PPI or not in 22 studies. Only Peng et al. showed a longer OS among patients with PPI (HR=1.22; 95%CI, 0.80–1.96). Overall, results showed that not using PPI significantly increased the OS of patients treated with ICI (HR=1.37; 95% CI, 1.23–1.52) (**Figure 2A**).

A statistically significant association was observed in 15 studies between PPI and ICI use and shorter PFS. However, in 15 studies, there was no difference in PFS whether patients treated with ICI received PPI or not. Only one study showed a benefit in PFS for patients treated with ICI and receiving PPI (HR=0.30; 95%CI, 0.10–0.70). Overall, results also showed that PFS was significantly and negatively associated with the use of PPIs with ICI treatment (HR=1.28; 95%CI, 1.15–1.42; **Figure 2B**).

**Figure 3:**
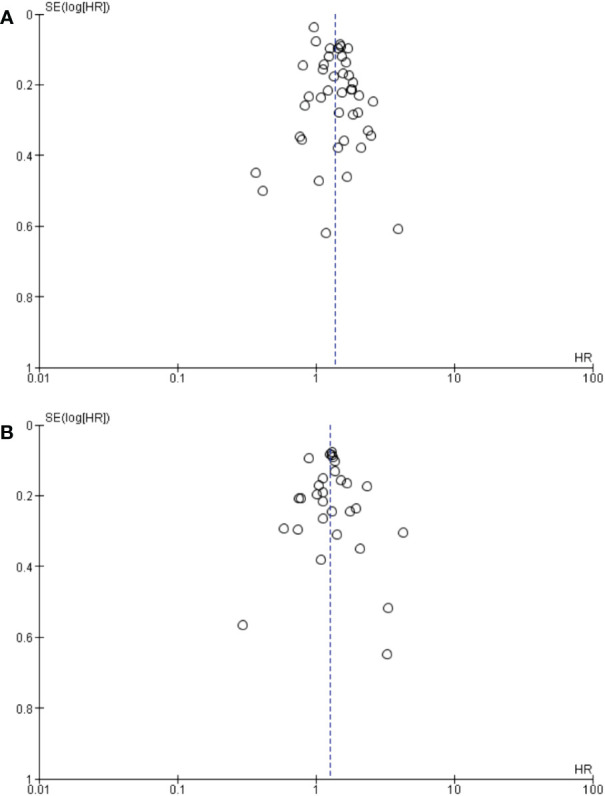
Begg’s funnel plot of HR ratios of **(A)** OS and **(B)** PFS. HR, hazard ratio; SE, standard error.

The between-study heterogeneity was moderate, with I² = 76% for OS and I² = 68% for PFS. Pooled HRs with 95% CIs were therefore calculated using random-effects models. The pooled HRs for OS were not significantly modified after excluding one study at a time in the sensitivity analysis. The pooled HRs for PFS did not significantly differ either in the sensitivity analysis (**Supplementary Tables S1**, **S2**). However, this did not necessarily seem relevant to analyze given the large number of studies included. There was no evidence of publication bias for pooled HR for OS and PFS analysis considering the funnel plots (**Figures 3A, B**).

**Figure 4:**
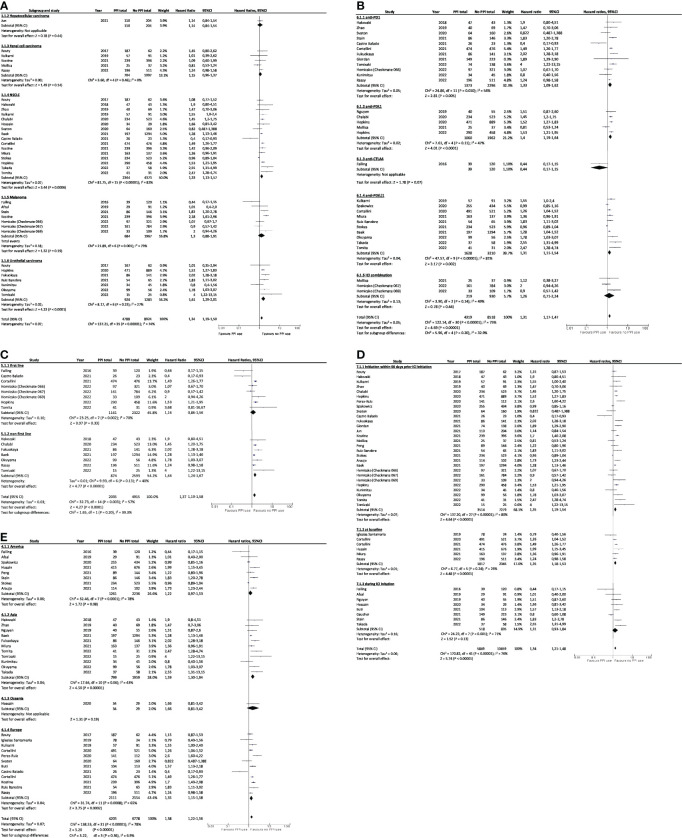
Subgroup analysis of association between PPI use and overall survival by **(A)** cancer type, **(B)** type of immunotherapy, **(C)** treatment line, **(D)** PPIs use window, and **(E)** continents. PPI, proton pump inhibitor; CI, confidence interval; NSCLC, non-small cell lung cancer; ICI, immune checkpoint inhibitor.

**Figure 5:**
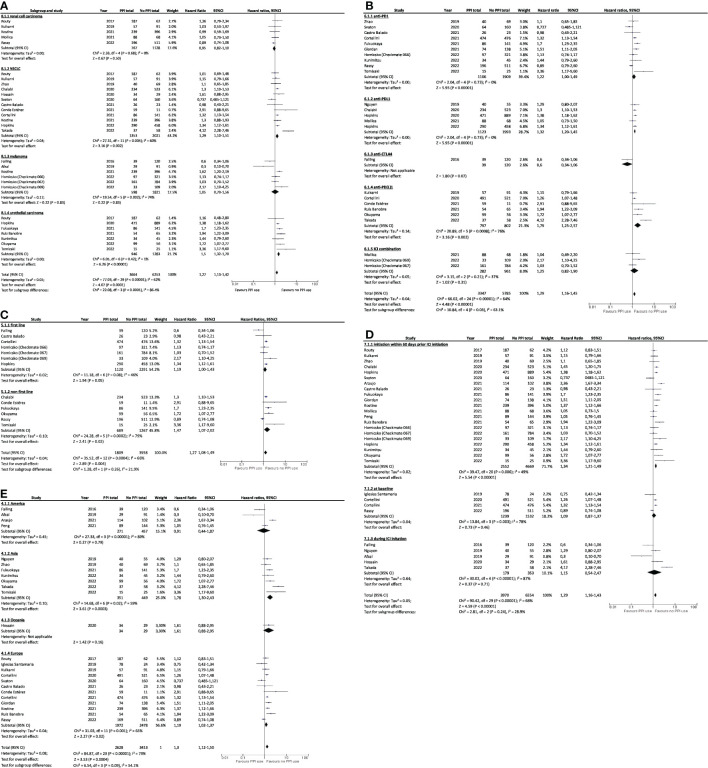
Subgroup analysis of association between PPI use and progression-free survival by **(A)** cancer type, **(B)** type of immunotherapy, **(C)** treatment line, **(D)** PPIs use window, and **(E)** by continents. PPI, proton pump inhibitor; CI, confidence interval; NSCLC, non-small cell lung cancer; ICI, immune checkpoint inhibitor.

Subgroup analysis showed that worst OS was associated with PPI and ICI use for NSCLC or urothelial cancer patients (HR=1.33, 95%CI 1.13–1.57 and HR=1.61, 95%CI 1.29–2.01; **Figure 4A**), and for patients treated with anti PD-1 and anti PD-L1 immunotherapies (HR=1.33, 95%CI 1.09–1.62 and HR=1.31, 95%CI 1.11–1.54, **Figure 4B**) when administered in non-first line (HR=1.44; 95%CI, 1.24–1.67; **Figure 4C**), and when PPIs was received as baseline treatment or in 60 days before ICIs initiation (HR=1.35, 95%CI 1.18–1.53 and HR=1.35, 95%CI 1.19–1.54; **Figure 4D**). OS was also worst for patients originating from Europe (HR=1.35; 95%CI, 1.15–1.58; **Figure 4E**) and Asia (HR=1.59; 95%CI, 1.30–1.94; **Figure 4E**). The lowest PFS were also associated with PPI and ICI use for NSCLC (HR=1.29; 95%CI, 1.10–1.51; **Figure 5A**) and urothelial carcinoma patients (HR=1.50; 95%CI, 1.32–1.70; **Figure 5A**), for patients treated with anti-PD-L1 (HR=1.32; 95%CI, 1.20–1.45; **Figure 5B**) when administered in non-first line (HR=1.47; 95%CI, 1.07–2.02; **Figure 5C**), and when PPIs were received in 60 days before ICIs initiation (HR=1.34; 95%CI, 1.21–1.49; **Figure 5D**). PFS was worst for Asian and European patients (HR=1.78, 95%CI 1.30–2.43 and HR=1.19, 95%CI 1.02–1.37; **Figure 5E**).

## 4 Discussion

Despite the revolution of cancer immunotherapies, the response rate of cancer patients to ICIs remains approximately 30% (56). Identifying predictive factors could contribute to improve patient selection for ICI treatment and is currently the topic of many ongoing research projects worldwide. Some predictive factors have already been identified but remain insufficient for patient selection in practice (e.g., PD-L1 expression, mutations, interferon signature). The present meta-analysis, which included 20,042 patients from 41 retrospective studies, suggested that concomitant PPI treatment was significantly associated with poorer OS and PFS in advanced solid cancer patients treated by ICIs. These results were in good agreement with the meta-analysis of Deng et al. (n = 16,147 patients, 30 publications) (57) and Chen et al. (n = 15,957 patients, 33 studies) (58). Two other meta-analyses showed no association between PPI consumption and survival outcomes in ICI patients (n=1,167 patients, five publications and 1,392 patients, seven studies, respectively) (59, 60), but a small sample size may have affected the results of the association between PPI use and ICI effectiveness.

Several studies showed that intestinal microbiota had a significant impact on immune system and ICI responses (61). *Bifidobacterium* sp., and in particular *B. breve*, *B. adolescentis* and *B. longum*, were associated with response to ICIs in mice models (14). Matson et al. (62) confirmed these data and observed other species in responders’ patients. In another study, Routy et al. (47) highlighted the abundance of *Akkermansia muciniphila*, *Ruminococcus* sp., *Alistipes* sp. in responders to ICIs. The oral supplementation of *A. muciniphila*, alone and/or with *Enterococcus hirae*, contributed to the restoration of the response to ICIs in mouse (47). However, a recent study showed that the best survival was achieved only when *A. muciniphila* was present in small quantities (median survival of 27.7 months compared to 7.8 months when present in large quantities and to 15.5 months when absent from the gastrointestinal tract) (63). In a Japanese study, *Clostridium butyrium* supplementations improved ICI efficacy for a cohort of NSCLC patients (54). Firmicutes, *Faecalibaterium prausnitzii*, *Streptococcus parasanguinis*, *Bacteroides caccae*, and high alpha diversity also appeared to be associated with patient’s response to ICIs, while Bacteroidetes, low alpha diversity, and *Escherichia coli* were associated with non-responder patients (64–66). Finally, T-cell response specific for *Bacteroides fragilis* was significantly associated with the response to anti CTLA-4 (67). The influence of the intestinal microbiome on the anticancer immune response varies depending on the species. Some bacteria found on patient’s responders treated with ICIs also showed different types of immune modulations, such as *B. fragilis*, which activated Th1 cells and cross-reactivity between bacterial antigens and tumor antigens (68). *Bifidobacterium* enhances interferon gamma (IFN-γ) production by TCD8 cells and the tumor infiltration, whereas *Akkermansia muciniphila* induces interleukin (IL)-12 (47). Microbiota was also shown to be involved in the activation of intratumoral and splenic dendritic cells (DC) (14). Gut microbiota microbial- or pathogen-associated molecular patterns (MAMPs or PAMPs) also participate to the immunomodulation of the tumor microenvironment. For example, lipopolysaccharides (LPS) improve adoptive T-cell activity (69), and bacterial DNAs modulate the balance of regulatory T/effective T cells (70). Finally, microbial metabolites can also be involved in the modulation of immune system (71). For example, short-chain fatty acids were shown to impact cytokines production, DC function (72, 73), and B-cell class switching and to facilitate Treg differentiation (74, 75).

PPI can also induce gut dysbiosis through their direct mechanism of action on HK/ATPase pump, which in turn reduces gastric acidity (76). More specifically, the population of bacteria associated with response to ICIs, including *Bifidobacterium* sp (76–78), Ruminococcaceae (76–78), *Akkermansia muciniphila* (79, 80), and *Alistipes* sp (77), were found to be decreased by PPI treatment. Alpha diversity was also negatively impacted by PPIs (75, 76, 81). On the contrary, the population of bacteria associated with resistance to ICIs, such as Bacteroidetes (79) and *Escherichia coli* (76, 77), increased with PPI treatment.

Obesity is a defined risk factor of GERD, and PPI are prescribed for GERD treatment. However, obesity seems to be correlated to better OS in patients treated by ICIs. In 2018, McQuade et al. showed a positive impact of high body mass index (BMI) on OS and PFS for metastatic melanoma patients treated by ICIs (82) but not for the chemotherapy group. In a 2022 retrospective study, Lee et al. showed similar results on OS for melanoma (83). In another study, Cortellini et al. (84) reported that PFS and OS were longer for patients with advanced cancers in BMI>25 group. Finally, in NSCLC patients treated by atezolizumab, survival was improved in the high BMI group (85). Several hypotheses to explain this association have been raised. First, obesity may cause low systemic inflammation and impaired immune response, which could induce exhausted T-cell (which expressed PD-1) and lymphocytes dysfunction (85). Leptin is more secreted, which could increase PD-1 expression too (83). Second, gut microbiota is modified in obese patients. Further research would, however, be necessary to verify these hypotheses. In previous cited publications, no possible confounder, such as concomitant treatment, was proposed, and it is therefore not clear if the population with high BMI had more or less PPIs than the normal BMI group. Similarly, retrospective studies that were investigating the association between PPIs and survival did not provide any information regarding BMI in PPI and PPI-free groups. BMI therefore seems to be a predictive factor of response to ICIs, but it is not possible to conclude on concomitant PPI treatment in the obese group. Further studies are recommended to more precisely evaluate the impact of PPI on this population.

Usually, patients in poor general condition may have more comedications, including PPI, which may explain the negative effect on OS or PFS. However, in the studies included in this meta-analysis, no difference in terms of ECOG score in PPI and PPI-free groups was observed. However, some other factors, such as comorbidities (e.g., cardiovascular, psychiatric, and gastrointestinal) or the number and sites of metastasis, were inconstantly presented, which could have induced some bias. Including these various factors would require a prospective study.

Results presented in this article are promising for better treatment strategies; however, meta-analyses present some limitations that somewhat reduce the scope of these results. First, the included publications were retrospective studies that could induce a selection bias and lead to some missing information. For example, the type of PPI use, dosage, duration, and timing of initiation were not available. In some studies, the concomitant medications were also not available, even though antibiotics are expected to have a deleterious impact on ICI outcomes. Some factors that may impact OS and/or PFS were also missing in several studies, such as PD-L1 expression, tumor mass, or LIPI score in lung cancer. Despite these few limitations, this meta-analysis was based on a strong conceptual framework, and the robustness of the main results were confirmed using sensitivity analyses. In addition, Higgins’s I² test was 72% for PFS and 65% for OS, indicating a substantial heterogeneity of the studies included in this meta-analysis. This heterogeneity can partly be explained by the different types of studies used for this meta-analysis, since *post-hoc* analyses of prospective studies, and retrospective studies and abstracts, were included. This was a voluntary choice to increase the number of patients included. Indeed, data on the subject are limited, and some of them lead to totally opposite conclusions without having any prospective study available to be able to conclude. Associating all the available data on the subject was therefore deemed interesting, even though this automatically resulted in the increase in the global heterogeneity. Consequently, the type of immunotherapy (monotherapy versus dual therapy) and associated treatment [such as anti-vascular endothelial growth factor (anti-VEGF)] and the type of PPI used could vary from one study to another. The studies may also have involved patients of different ethnicities, the time between the introduction of PPI and immunotherapy may have varied from one study to another, and the studies involved various types of cancers (such as NSCLC, melanoma, and urothelial), which prognosis varies with immunotherapy treatment independently of concomitant treatments. However, when available, PFS and OS data for bi-immunotherapy were always preferred to monotherapy data, and NSCLC cancer data were chosen over other cancer types in case of *post-hoc* analyses to reduce the heterogeneity. Similarly, multivariate data were systematically chosen over univariate data. While most of the studies included in this meta-analysis independently concluded that PPIs had a detrimental effect on survival, some showed opposite results (i.e., a beneficial effect on OS or in PFS) (17, 25). These studies all have in common that they were conducted on a relatively small sample, with <100 patients for each treatment arm and low statistical weight in the global meta-analysis. Subgroup analysis was therefore conducted to estimate whether the influence of PPI on different groups of interest (region, type of cancer, type of ICI, treatment line, and timeframe of PPI exposure). In the cancer-type subgroup, PPI use was significantly associated with a significant decrease in OS and PFS for NSCLC and urothelial carcinoma. On the opposite, PPI use was associated (yet not statistically significantly) with improved PFS among patients with melanoma. This observation could (at least partially) be explained by the fact that patients with melanoma are often treated with anti-CTLA4 and that in the ICI type subgroup, patients treated with anti-CTLA4 had greater PFS and OS outcome when concomitantly treated with PPIs. However, only one study was included in the CTLA4 subgroup (Failing 2016), so more retrospective studies are needed to clarify the role of each subgroup on the correlation between PPIs and ICIs. In addition, the plan was initially to additionally investigate the effect of ECOG status and age, but there were too many missing data to conclude.

In conclusion, the meta-analysis conducted in this research suggested that concomitant PPI treatment was significantly associated with poorer OS and PFS for advanced solid cancer patients treated with ICIs. The evaluation of PPI necessity and indication by clinicians is therefore strongly recommended at the initiation of anticancer immune therapies. PPI deprescription should be conducted whenever possible, following deprescribing protocols (86, 87). Information concerning the type of PPI use, the posology, the duration, and the moment of their initiation should systematically be reported to improve future retrospective analyses. Larger prospective studies adjusting for cofounding factors are needed to determine the reel impact of PPI on survival outcomes in patients treated by ICIs and to evaluate the time limit of initiation and posology impact. A follow-up of microbiota changes in patients treated concomitantly with PPIs and ICIs would also be useful to determine the moment when negative impact of PPIs may appear.

## Data availability statement

The original contributions presented in the study are included in the article/**Supplementary Material**. Further inquiries can be directed to the corresponding author.

## Author contributions

There are two first authors in this manuscript, SL and LP, who worked equally to this project. LP was responsible of statistical analyses. MK and SL granted the quality assessment of the included studies. SL and LP were responsible for data analysis and writing the article. CM, AD, BM, and BG were responsible for the design of the project. All authors contributed to the article and approved the submitted version.
