# Supplementary material for: Do proton pump inhibitors alter the response to immune checkpoint inhibitors in cancer patients? A meta-analysis
Source: Front Immunol. 2023 Jan 26;14:1070076. doi: 10.3389/fimmu.2023.1070076 (PMC9910608; doi:10.3389/fimmu.2023.1070076)
Supplement: Supplementary file 1 [file DataSheet_1.docx]

Supplementary Material

Do Proton Pump Inhibitors alter the response to immune checkpoint inhibitors in cancer patients? A meta-analysis

**Lopes Sébastien**, **Pabst Lucile***,* **Dory Anne**, **Klotz** **Marion, Gourieux Bénédicte**, **Michel Bruno**, **Mascaux Céline***

*** Correspondence:** Celine Mascaux. e-mail: [celine.mascaux@chru-strasbourg.fr](mailto:celine.mascaux@chru-strasbourg.fr)

# Supplementary Figures and Tables

## Supplementary tables

Supplementary table 1: Sensitivity analysis for Overall Survival

| Study removed | HR | 95%CI |
| --- | --- | --- |
| Afzal 2019 | 1,37 | 1,23-1,53 |
| Araujo 2021 | 1,36 | 1,22-1,51 |
| Baek 2021 | 1,36 | 1,22-1,51 |
| Buti 2021 | 1,36 | 1,22-1,52 |
| Castro Balado 2021 | 1,39 | 1,25-1,54 |
| Chalabi 2020 | 1,37 | 1,22-1,52 |
| Cortellini 2020 | 1,37 | 1,23-1,53 |
| Cortellini 2021 | 1,36 | 1,22-1,52 |
| Failing 2016 | 1,38 | 1,25-1,54 |
| Fukuokaya 2021 | 1,36 | 1,22-1,51 |
| Gaucher 2021 | 1,39 | 1,25-1,55 |
| Giordan 2021 | 1,36 | 1,22-1,51 |
| Hakozaki 2018 | 1,37 | 1,23-1,52 |
| Homicsko (Checkmate 066) 2022 | 1,38 | 1,24-1,53 |
| Homicsko (Checkmate 067) 2022 | 1,38 | 1,24-1,54 |
| Homicsko (Checkmate 069) 2022 | 1,36 | 1,22-1,51 |
| Hopkins 2020 | 1,36 | 1,22-1,52 |
| Hopkins 2022 | 1,36 | 1,22-1,52 |
| Hossain 2020 | 1,37 | 1,23-1,52 |
| Husain 2021 | 1,36 | 1,22-1,51 |
| Iglesias Santamaria 2019 | 1,38 | 1,24-1,54 |
| Jun 2021 | 1,38 | 1,24-1,53 |
| Kostine 2021 | 1,36 | 1,22-1,51 |
| Kulkarni 2019 | 1,36 | 1,23-1,52 |
| Kunimitsu 2022 | 1,38 | 1,24-1,53 |
| Miura 2021 | 1,37 | 1,23-1,53 |
| Mollica 2021 | 1,37 | 1,23-1,52 |
| Nguyen 2019 | 1,37 | 1,23-1,52 |
| Okuyama 2022 | 1,36 | 1,22-1,51 |
| Peng 2021 | 1,37 | 1,23-1,53 |
| Perez-Ruiz 2020 | 1,35 | 1,22-1,50 |
| Rassy 2022 | 1,37 | 1,23-1,53 |
| Routy 2017 | 1,38 | 1,24-1,54 |
| Ruiz Banobre 2021 | 1,36 | 1,22-1,51 |
| Spakowicz 2020 | 1,39 | 1,24-1,55 |
| Stein 2021 | 1,36 | 1,22-1,51 |
| Stokes 2021 | 1,39 | 1,26-1,53 |
| Svaton 2020 | 1,38 | 1,24-1,54 |
| Takada 2022 | 1,36 | 1,22-1,51 |
| Tomita 2022 | 1,36 | 1,22-1,51 |
| Tomizaki 2022 | 1,36 | 1,22-1,51 |
| Zhao 2019 | 1,37 | 1,23-1,52 |

Abbreviation: HR, hazard ratio; CI, confidence interval

Supplementary table 2: Sensitivity analysis for Progression Free Survival

| Study removed | HR | 95%CI |
| --- | --- | --- |
| Araujo 2021 | 1,25 | 1,13-1,38 |
| Takada 2022 | 1,25 | 1,13-1,38 |
| Fukuokaya 2021 | 1,26 | 1,13-1,41 |
| Ruiz Banobre 2021 | 1,26 | 1,13-1,40 |
| Hossain 2020 | 1,27 | 1,14-1,42 |
| Hopkins 2020 | 1,27 | 1,14-1,42 |
| Conde Estérez 2021 | 1,27 | 1,14-1,41 |
| Giordan 2021 | 1,27 | 1,14-1,41 |
| Kostine 2021 | 1,27 | 1,14-1,42 |
| Homicsko (Checkmate 069) 2022 | 1,27 | 1,14-1,41 |
| Hopkins 2022 | 1,27 | 1,14-1,43 |
| Kunimitsu 2022 | 1,27 | 1,14-1,42 |
| Okuyama 2022 | 1,27 | 1,14-1,41 |
| Tomizaki 2022 | 1,27 | 1,14-1,41 |
| Routy 2017 | 1,28 | 1,15-1,43 |
| Kulkarni 2019 | 1,28 | 1,15-1,43 |
| Nguyen 2019 | 1,28 | 1,15-1,42 |
| Zhao 2019 | 1,28 | 1,15-1,43 |
| Chalabi 2020 | 1,28 | 1,14-1,43 |
| Cortellini 2020 | 1,28 | 1,14-1,43 |
| Cortellini 2021 | 1,28 | 1,14-1,43 |
| Mollica 2021 | 1,28 | 1,15-1,43 |
| Homicsko (Checkmate 066) 2022 | 1,28 | 1,15-1,43 |
| Afzal 2019 | 1,29 | 1,16-1,43 |
| Iglesias Santamaria 2019 | 1,29 | 1,16-1,44 |
| Peng 2021 | 1,29 | 1,16-1,43 |
| Homicsko (Checkmate 067) 2022 | 1,29 | 1,16-1,43 |
| Failing 2016 | 1,3 | 1,17-1,44 |
| Svaton 2020 | 1,3 | 1,17-1,44 |
| Castro Balado 2021 | 1,3 | 1,17-1,44 |
| Rassy 2022 | 1,3 | 1,18-1,44 |

Abbreviation: RR, risk ratio; CI, confidence interval
